# Preliminary Feasibility Implementation of Post-Decompressive Craniectomy 3D-Printed Externally Applied Cranial Orthosis (PEACO) During Inpatient Rehabilitation: A Retrospective Cohort Study Reporting Short-Term Tolerability and Patient/Caregiver-Reported Feedback

**DOI:** 10.3390/healthcare14203361

**Published:** 2026-10-09

**Authors:** Karen Sui Geok Chua, Ravi Shankar, Michael Gui Jie Yam, Emily Yee, Rathi Ratha Krishnan, Tegan Kate Plunkett, Wei Binh Chong, Jaclyn Ai Mei Low, Nasrul Hadi B. Said, John Chao, Suan Gek Ng, Jai Prashanth Rao

**Affiliations:** 1 Institute of Rehabilitation Excellence (IREx), Tan Tock Seng Hospital Rehabilitation Centre, National Healthcare Group Health, 1 Tan Tock Seng Link, Singapore 307382, Singapore; karen.chua@nhghealth.com.sg (K.S.G.C.); emily_yee@nuhs.edu.sg (E.Y.); rathi.ratha.krishnan@nhghealth.com.sg (R.R.K.); tegan.kate.plunkett@nhghealth.com.sg (T.K.P.); wei.binh.chong@nhghealth.com.sg (W.B.C.); jaclyn.am.low@nhghealth.com.sg (J.A.M.L.); 2 R3 Institute Singapore (R3I), Nanyang Technological University, 11 Mandalay Road, Singapore 308232, Singapore; michael.yam@nhghealth.com.sg; 3 Yong Loo Lin School of Medicine, National University of Singapore, 10 Medical Drive, Singapore 117597, Singapore; jai.rao@singhealth.com.sg; 4 Clinical Research and Innovation Office, Tan Tock Seng Hospital, National Healthcare Group Health, 11 Jln Tan Tock Seng, Singapore 308433, Singapore; 5 3D Medical Printing Centre, Tan Tock Seng Hospital, National Healthcare Group Health, 11 Jln Tan Tock Seng, Singapore 308433, Singapore; nasrul@medairum.com (N.H.B.S.); yuan-yu.chao@nhghealth.com.sg (J.C.); 6 Department of Orthopaedic Surgery, Tan Tock Seng Hospital, National Healthcare Group Health, 11 Jln Tan Tock Seng, Singapore 308433, Singapore; 7 Medairum Pte Ltd., 701 Sims Drive, LHK Building, Singapore 387383, Singapore; 8 Department of Neurosurgery, National Neuroscience Institute, Singapore Health Services, 11 Jln Tan Tock Seng, Singapore 308433, Singapore; ng.suan.gek@singhealth.com.sg; 9 Duke-NUS Medical School, 8 College Road, Singapore 169857, Singapore

**Keywords:** 3D-printed, cranial orthosis, decompressive craniectomy, stroke, traumatic brain injury, rehabilitation

## Abstract

**Highlights:**

**What are the main findings?**

- The authors describe a detailed preliminary clinical implementation pathway conducted during inpatient brain injury rehabilitation. A minimally monitored inpatient PEACO clinical protocol achieved 18/23 (78.3%) compliance without serious adverse events (e.g., wound dehiscence/infection) across 23 decompressive craniectomy patients.
- Successful integration within established tertiary centre workflows without additional personnel for monitoring, provides the initial framework for future studies on broader scalability, implementation and future evaluation of economic impact or cost-effectiveness.

**What are the implications of the main findings?**

- Short-term feasibility, tolerability, and absence of observed serious device-related complications of PEACO were demonstrated within an embedded preliminary clinical rehabilitation workflow.
- Larger, multicentre studies with longer follow-up are needed to confirm cranial protection safety and sustained compliance to inform future clinical practice guidelines for post-decompressive craniectomy (DC) cranial protection.

**Abstract:**

A minimally monitored clinical protocol demonstrated preliminary short-term feasibility and tolerability: 18/23 (78.3%) fitted patients were confirmed compliant, with 18/20 (90%) respondent compliance and three patients of unknown status, without serious adverse events. Longer follow-up studies are needed to assess sustained compliance throughout the pre-cranioplasty period. Future research should standardise materials and outcome measures to inform clinical practice guidelines. **Background/Objectives**: 3D-printed externally applied cranial orthosis (PEACO) represents an emerging solution for temporary cranial protection, though evidence remains limited regarding real-world implementation using minimally monitored clinical protocols. **Methods**: Retrospective cohort study conducted at a 121-bed rehabilitation centre in Singapore (November 2022–November 2025). Adults >21 years, >3 weeks post-DC with healed wounds and available caregivers were included. PEACO devices were individually designed from CT stereotaxis scans using contralateral hemi-cranium mirroring and fabricated using Ultra-durable Resin. Rehabilitation therapists fitted devices using progressive wearing schedules. T0 outcomes (30 min post-fitting) included therapist-observed pain, redness, skin imprints, and pruritus. T1 outcomes (at least 2 weeks post-fitting) included wearing duration, cosmesis ratings (0–10 scale), compliance, and adverse events via digital surveys. Exploratory comparisons using descriptive analysis were performed to compare outcomes from 2021 (*n* = 10) and 2025 (*n* = 23) cohorts. **Results**: Twenty-three patients (14 males, median age 56.0 years) were fitted with PEACO at median 67 days post-DC, (13 traumatic brain injury, 10 stroke). At T0, primary reprinting for poor fit was required in 3/23 (13.0%), 18/23 (78.3%) fitted patients were confirmed compliant, 18/20 (90%) were compliant with responder feedback; 2/20 (10.0%) were non-compliant, with three patients of unknown status. At T0, 9/23 (39.1%) had therapist-observed mild, transient adverse events, with no reported wound dehiscence. At T1, among 18 respondents: 16/18 (88.9%) wore PEACO ≤4 h/day, 7/18 (38.9%) reported self-limiting discomfort or transient skin changes at T1, median cosmesis rating was 6.0/10. At T1 assessment, no falls with head impact occurred. Clinical Trials Registration: ClinicalTrials.gov NCT07122752.

## 1. Introduction

3D printing technology applications for healthcare have shown rapid advances in materials, production time, biomechanical improvements, and comfort in relation to wearable lower and upper limb orthosis and therapeutic cranial moulding helmets. However, the current literature suggests that 3D-printed devices may come with higher costs and longer production times. Moreover, demonstrable, or significant changes in clinical outcomes between 3D-printed versus traditional orthoses remain uncertain and relative gaps in reporting of clinical outcomes are acknowledged [1].

With regard to decompressive craniectomy (DC), this refers to life-saving surgery performed for severe strokes or traumatic brain injuries (TBI) [2,3]. DC survivors may suffer secondary complications such as secondary TBI from falls related to impaired balance and gait or risk-taking behaviour from cognitive impairment, epilepsy, and syndrome of the trephined brain [4]. The latter complication is of concern as it can potentially cause further neurological decline and mortality due to paradoxical herniation and cortical compression from the sinking brain flap [4]. Reversal of these conditions is only possible via subsequent cranioplasty, using either autologous bone or artificial materials [5].

The optimal timing of elective cranioplasty remains controversial [6,7], with the need to balance infection risks which increase with a short duration to cranioplasty (<14 days post-event); while new-onset complications such as delayed seizures are increased (>90 days); longer operating time (>12 weeks); while better motor and cognitive outcomes are associated with earlier cranioplasty (<90 days) [2,3]. For most DC patients, a 3 to 6 month wait for definitive cranioplasty is not uncommon.

During this period, DC patients are prone to a variety of complications such as late- onset epilepsy, syndrome of the trephined brain, paradoxical herniation, haemorrhage, and post-surgical infections, in addition to trauma vulnerability and flap herniation from the cranial defect [8,9]. Despite the importance ascribed to the need for mandatory helmet use during rehabilitation, the literature is scarce regarding the optimal management of the DC skull defect prior to cranioplasty [10]. Furthermore, an estimated 65% of staff did not implement preventive protective measures for the cranial defect during patient transfers or positioning [11].

3D-printed externally applied cranial orthosis (PEACO) is an emerging trend towards temporary cranial protection offering better anatomical fit, customisation, compliance, and superior wearability. The recent literature provides the fundamental evidence for device designs and 3D printing materials with initial trials reporting high usability and acceptance with minimal adverse events [12]. To date, the majority of PEACO were deployed within closely monitored protocols over 3-to-6-month periods [12].

The aims of this feasibility study were to: (i) describe the clinical inpatient protocol for PEACO, (ii) report short-term tolerability, acceptability, and outcome of PEACO within 30 min of fitting (T0), and (iii) at 2 weeks post-fitting by patient-reported outcome measures, using a minimally monitored protocol (T1). Secondarily, we compared T1 outcomes of current version (from 2022), with those of a previous experimental version (2021) [13].

## 2. Materials and Methods

### 2.1. Study Design and Setting

A retrospective cohort study was conducted from 11 August 2025 to 30 January 2026 at a single research site, the Tan Tock Seng Hospital (TTSH) rehabilitation centre, Integrated Care Hub, Singapore, a 121-bed inpatient rehabilitation facility with 20 dedicated acquired brain injury rehabilitation unit beds, which has direct links to an acute neurotrauma centre. Data were derived from audit outcomes of a pilot clinical cranial cap service, in acute DC patients, collected by rehabilitation therapists between 15 November 2022 and 20 November 2025. Patient feedback data were consecutively recruited, and the current 2022 PEACO version was used. (Refer to File S1, Figure S1 for the study chronology).

### 2.2. Ethics Statement

Ethics approval was obtained from National Healthcare Group, Domain Specific Review Board (NHG DSRB 2025-1218, date of approval 11 August 2025, approver Dr Chen Wen Kai; Refer to File S1). A waiver of consent was obtained as the study was related to a clinical service audit, wherein recontacting discharged inpatients would be difficult. No patient identifiers were retained in the final dataset. All data collection procedures were performed by research assistants in accordance with the Declaration of Helsinki, WHO. The study was registered with ClinicalTrials.gov (NCT07122752).

### 2.3. Features of PEACO

**Figure 1:**
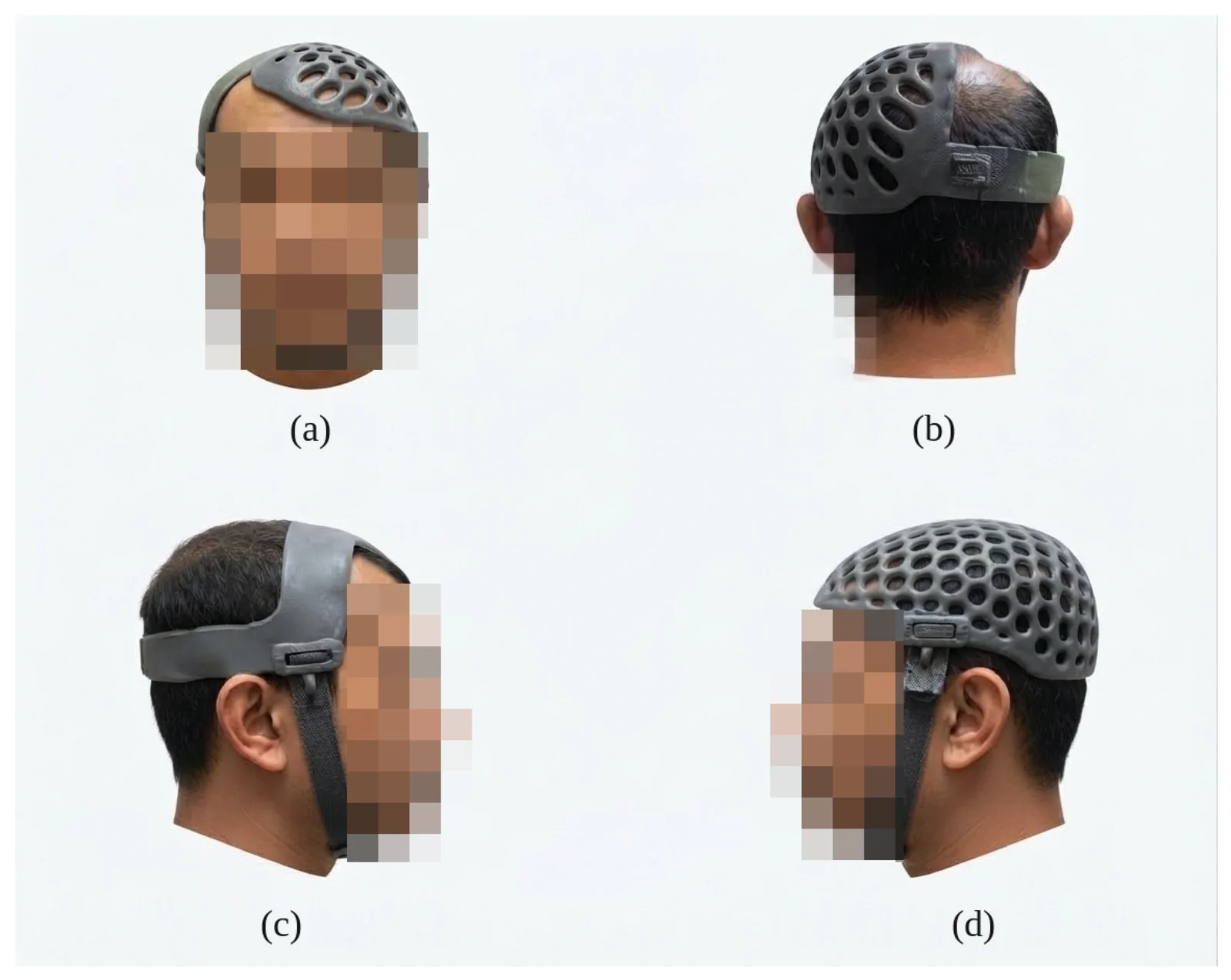
3D-printed cranial cap: (**a**) Frontal View; (**b**) Back View; (**c**) Right Lateral View; (**d**) Left Lateral View. Facial features have been masked to protect anonymity.

All devices in this study were designed and manufactured at the 3D Medical Printing Centre, TTSH by a single staff (NHBS) using standardised materials and design parameters. Key clinical design features of the current PEACO model included: (i) a perforated protective layer over the cranial defect; (ii) integrated single-piece strap for stable, one-handed independent self-application; (iii) posterior and superior strap placement for improved stability; (iv) cap thickness of 3 mm with rounded edges; (v) patient-specific contouring; and (vi) chinstraps added after printing. Figure 1a–d illustrate the four device views.

The current version used Ultra-durable Resin (Formlabs, Tough 1500 Resin V2, FLTO1502; Formlabs, Somerville, MA, USA) to improve impact resistance, as impact absorption was prioritised over tensile loading for cranial protection. This material was selected for its high impact resistance (unnotched Izod 910 J/m; notched Charpy 7.5 kJ/m^2^ post-cured) despite moderate tensile strength (34 MPa) [14].

No device specific mechanical testing nor drop testing was performed. Formlabs Tough 1500 Resin V2 (Formlabs, Somerville, MA, USA) was printed on a Form 4B/4BL SLA printer (Formlabs, Somerville, MA, USA). Note: the current manufacturing workflow using Form 4B/4BL printers (available from 2024) and Tough 1500 Resin V2 (available from March 2025) applies to devices fabricated from 2025 onwards. Devices fabricated between November 2022 and 2024 used earlier-generation Formlabs printers (Form 3B/3BL; Formlabs, Somerville, MA, USA) and Tough 1500 Resin V1 (Formlabs, Somerville, MA, USA). De-identified manufacturing records detailing the fabrication period, printer model, resin version, and relevant design changes for each device analysed, including reprints, are available from the corresponding author upon reasonable request. The material was chosen for the following justification reasons: resin is characterised by three times higher Gardner impact strength for thin walls that do not shatter, compared to the previous material version, along with high elongation at break and flex fatigue resistance. Its ultimate tensile strength is 33.0 MPa and tensile modulus is 150.0 MPa (per ASTM D638-14 [15]), with 51% elongation at break, and a Notched Izod impact value of 67.0 J/m. Formlabs characterises the material as offering strength, stiffness, and toughness comparable to polypropylene (PP), with exceptional resistance to fracture, impact, and shattering—mechanical behaviour consistent with materials widely used in impact-rated protective equipment (e.g., PP/PE-based helmet shells). This provides a reasonable basis to expect adequate energy absorption and resistance to brittle fracture under incidental impact loading [14].

Regarding the curved design, this was necessary as the cap geometry is patient-specific, digitally derived from the patient’s cranial anatomy to produce a smooth, continuously curved, low-profile shell (nominal thickness 4 mm, minimum 3 mm at the thinnest section). The curved, domed geometry is inherently more resistant to localised impact than a flat panel of equivalent thickness: curvature promotes distribution of impact load across the shell via membrane/arch action, reducing peak local stress and deflection at the point of contact, and avoids flat or sharply angled zones that would otherwise concentrate stress. This was analogous to the load-distributing mechanism relied upon in conventional dome-shaped protective helmets. Regarding ventilation holes, these were 10 mm diameter and associated with 15 mm minimum clear spacing between holes with minimum 10 mm edge distance from cap perimeter/boundary. These were incorporated for patient comfort, optimal cap mass, and thermoregulation. Hole placements were distributed across the mid-region of the cap and kept clear from the edge of the cap which overlaid the craniectomy defect. This design of specified edge distance and inter-hole spacing preserved a continuous load path of solid material around each perforation, limiting local stress concentration and maintaining structural continuity of the shell, given the wall thickness (3–4 mm) relative to hole diameter (10 mm), the perforated region is not considered to substantially compromise the overall impact-distributing capacity of the curved shell. Collectively, these design choices are consistent with established engineering principles applicable to protective shell structures [14]. However, this study does not evaluate the impact attenuation or protective performance of the complete PEACO device, and the above considerations remain theoretical without device-level mechanical testing.

In comparison with the experimental model (2021) [13], several design elements were refined. First, the circumferential horizontal strap was replaced with an integrated right-angled strap connected directly to the cranial piece, providing additional stability during head displacement and enabling independent donning and doffing for patients with stroke-related hemiplegic arm weakness. A single strap was added post-production; it was deemed not to have effects on the PEACO integrity.

The custom cranial cap manufacturing process began with computed tomography acquisition, the parameters of which have been provided, followed by segmentation of the resulting imaging data in 3D Slicer (open-source software; www.slicer.org) and subsequent digital design refinement in Meshmixer (Autodesk, San Francisco, CA, USA; www.meshmixer.com). The finalised model was printed on a Form4BL using the preset settings validated for Formlabs Tough 1500 V2, oriented with the inner surface of the cap facing upward such that support structures are generated only on the external surface, at a layer thickness of 100 microns. Post-processing consisted of washing in 99 percent isopropyl alcohol for 20 min, followed by curing in a Form Cure L unit (Formlabs, Somerville, MA, USA) for 60 min at 70 degrees Celsius. Quality control on the 3D printing side is performed by 3D scanning the completed cap and comparing it against the original digital file using CloudCompare (open-source software; www.cloudcompare.org), with an allowable surface deviation of up to 1 percent; additionally, the patient identifier printed on an accompanying 15 by 25-millimetre reference block is verified by digital calliper measurement against the same 1 percent tolerance. The device was classified as a custom device, and the Tough 1500 resin used in its fabrication has been evaluated as a skin-contacting material in accordance with ISO 10993-1 [16], having passed the requirements of ISO 10993-5 [17] for cytotoxicity, ISO 10993-10 [18] for irritation, and ISO 10993-10 [18] for sensitization. (Please refer to File S2 for CT parameter acquisition).

### 2.4. Patients Criteria During Clinical Pilot Service

Inclusion criteria were adults aged >21 years, >3 weeks post-DC, with healed surgical wounds and available carers. All aetiologies of DC were included (e.g., stroke, traumatic brain injury, brain tumours or infections). Exclusion criteria included bulging cranial flaps, unhealed surgical wounds, and disorders of consciousness (DoC). The latter were not offered PEACO due to their dependent physical status being bed bound, minimal risk of fall, reduced need for cranial protection, and inability to report wearability feedback.

### 2.5. Clinical Protocol

**Figure 2:**
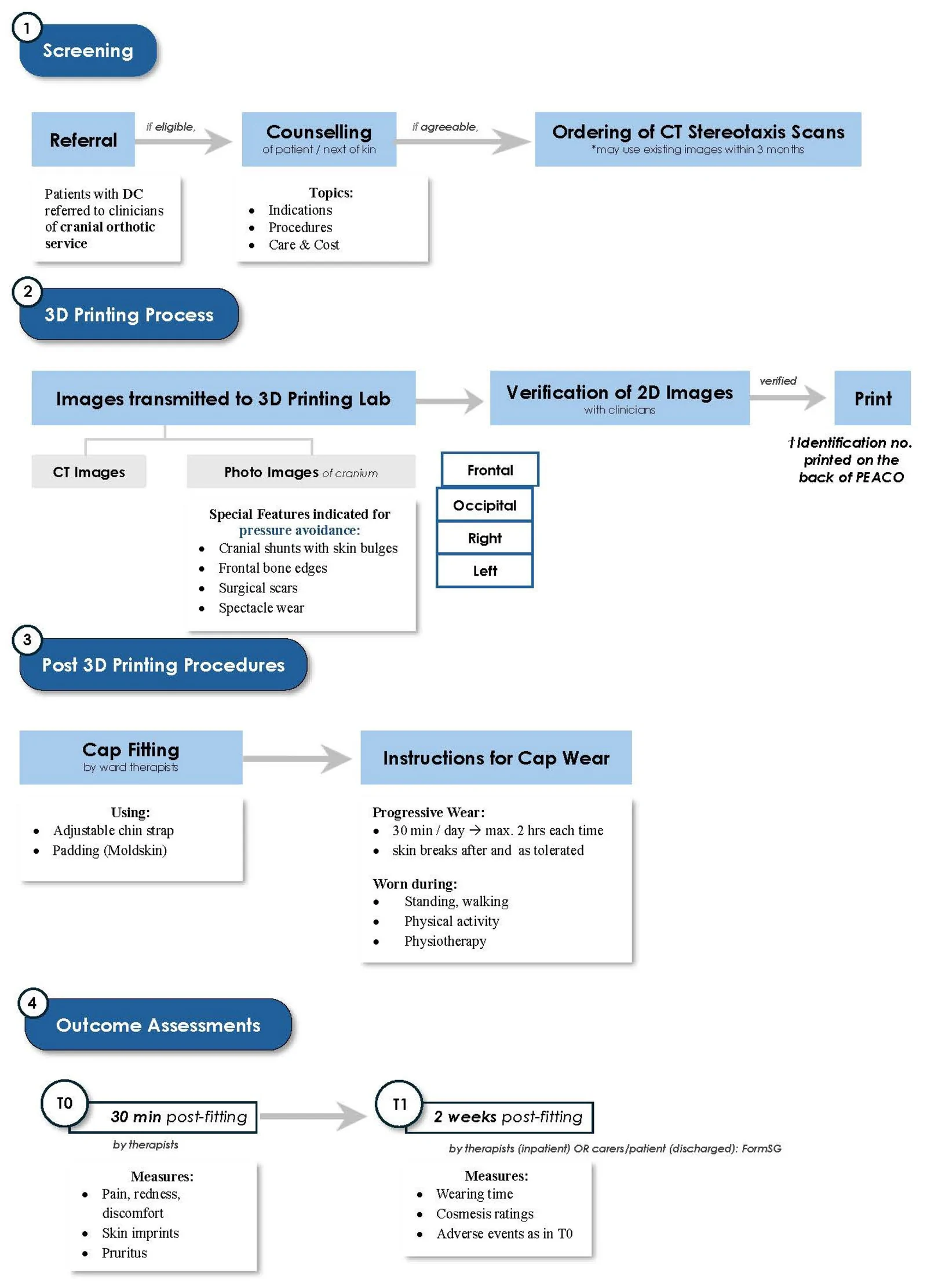
A workflow diagram showing the complete PEACO clinical process from screening through follow-up, with team responsibilities indicated. Notes in the diagram are indicated by the following symbols: asterisk (*): existing images acquired within the preceding 3 months may be used for the CT stereotaxis scans; dagger (†): an identification number is printed on the back of the PEACO.

Figure 2 summarises the clinical workflow for all inpatients for whom PEACO was prescribed. All patients with DC were referred to clinicians of the cranial orthotic service, who assessed eligibility. Eligible patients and their next of kin were counselled on indications, processes, and care instructions (refer to File S3, Figures S2 and S3); verbal consent was obtained and documented in the patients’ electronic medical records. CT stereotaxis brain scans were then ordered electronically. Photo images of four views (frontal, occipital, right and left) of the patient’s cranium were obtained and special skin features for pressure avoidance were transmitted via a secure electronic ordering Form.sg (www.form.gov.sg) to technicians at the 3D printing lab.

In terms of the 3D printing process, CT stereography images were obtained, and segmentation of the patient’s skull was performed to show the craniectomy defect. Mirroring of the contralateral hemi-cranium was performed to ensure symmetric shape. A customised cranial cap was designed with standard design parameters and printed on the Formlabs4B in Ultra-durable Resin. Post-processing included removal of support material, washing and curing. Post-printing quality checks were performed manually and digitally, to ensure print accuracy. Subsequently, PEACO was hand-delivered to the patients’ rehabilitation wards by research assistants. Moldskin (Molnlycke Health Care, Gothenburg, Sweden) self-adhesive cushioning was added as needed, to the inner aspect for comfort and cushioned frontal bone, off-loading effect by therapists. Finally, adjustable chin straps using hook-and-loop (Velcro Companies, Manchester, NH, USA) fasteners were clipped onto the customised right-angled rigid straps and buckle and sized accordingly.

Progressive wearing commenced at 30 min/day with the completed PEACO and increased by 30 min daily to a maximum of 2 h continuous wear every 24–48 h as tolerated. PEACO was worn during periods of standing, walking, physical activity in the wards and during rehabilitation therapies. Patients were encouraged to remove their caps after 2 h of continuous wear to allow for skin ventilation or if any skin discomfort, pain or skin changes were observed. Patients and their carers were discharged with written care instructions, and a phone hotline number was given in case of subsequent adverse events. The billed cost of PEACO was SGD 650/- and this was charged to the inpatient hospital bill.

### 2.6. Outcome Assessment

Relevant implementation outcomes such as successful first fitting, reprinting due to technical failure, modification rates, fabrication time, time to delivery, staff workload, required training, and device cost.

As part of clinical service audit, clinical outcomes were assessed at 2 timepoints; by therapists at T0 (30 min post-fitting), and at T1 (>2 weeks post-fitting) by patients and/or caregivers while inpatient or by telephone if discharged, using secure digital Form.sg (www.form.gov.sg) administered by research assistants (Please refer to File S4, Figure S4). Due to residual cognitive/communication impairment following DC, which could affect patients’ feedback, we allowed both patients’ and caregivers’ responses at T1.

At T0, patients were observed after 30 min of continuous PEACO wear for adverse events such as pain, discomfort or pruritus and signs of redness, skin imprints (termed mild adverse events) or wound dehiscence (serious adverse events). At T0, a minimum two-week follow-up interval was selected as most device-related adverse events were anticipated during this initial adaptation phase. Furthermore, a shorter recall period was chosen for the following reasons; it provided enhanced reliability of patient-reported outcomes in cognitively impaired populations, acceptability to carers and staff as compared to a prolonged monitoring period as no additional staff were employed for this, and previous experience with the high safety and durability profile of the experimental prototype (2021) [13]. T1 measures included surrogate measures of reported device use (as distinct from adherence to the prescribed wearing schedule) comprising: (a) wearing time (classified as 0–1 h, 2–4 h, and >4 h/day, consistent with the original questionnaire categories); (b) cosmesis ratings (Likert scale 0–10; 0 as unacceptable and 10 as very acceptable); and (c) adverse events (presence/absence of pain, discomfort (skin comfort visual analogue rating 0 (not comfortable)–10 (very comfortable)), pruritus, skin imprints/breakdown).

Due to refinements in data collection protocols between the experimental prototype (2021) and current (from 2022) clinical model deployment periods, we harmonised these 2 models’ disparate adverse event terminology to enable meaningful but exploratory comparisons. At T0: “Redness” and “Imprints” (current) were both matched to “Skin Changes” (experimental). At T1: “Discomfort” (current) was matched to “Pressure” (experimental), “Itch” (current) to “Itch” (experimental), and “Skin Markings” (current) to “Skin Changes” (experimental) [13].

### 2.7. Data Collection

Data regarding demographics, injury, rehabilitation data (Functional Independence Measure [FIM] [19], acute and rehabilitation length of stays, duration to PEACO fitting) and PEACO mass (g) were extracted from discharged electronic medical records and combined with T0 and T1 data to create an electronic database.

### 2.8. Statistical Analysis

In summary, all variables were extracted from electronic medical records without retention of identifiers and combined with T0, T1 outcome data to form the final dataset. Statistical analyses were performed using R version 4.2.0 (R Foundation for Statistical Computing, Vienna, Austria), while R Studio (Posit PBC, Boston, MA, USA; www.posit.co) used as the integrated development environment. A total of 23 participants were included in the analysis. Missing data management used analyses conducted using available-case data, with denominators reflecting the number of participants with available data for each outcome. Missing observations were not imputed. Descriptive statistics were calculated for all variables, with continuous variables presented using median and interquartile range (IQR) due to non-normal distribution, while categorical variables were presented using frequencies and percentages, *n* (%).

Comparisons between the 2 data groups (current and experimental) were performed using Fisher’s exact test for categorical variables and Mann–Whitney U test for continuous variables. Statistical significance was set at *p* < 0.05 for all analyses.

The 95% confidence intervals for the main proportions and effect estimates for comparisons between the experimental and current cohorts are displayed in File S5, Table S1. Given the small sample sizes and technical and methodological differences requiring variable harmonisation, the comparison between both groups is interpreted as exploratory and hypothesis-generating rather than confirmatory.

## 3. Results

### 3.1. Baseline Data (T0)

**Figure 3:**
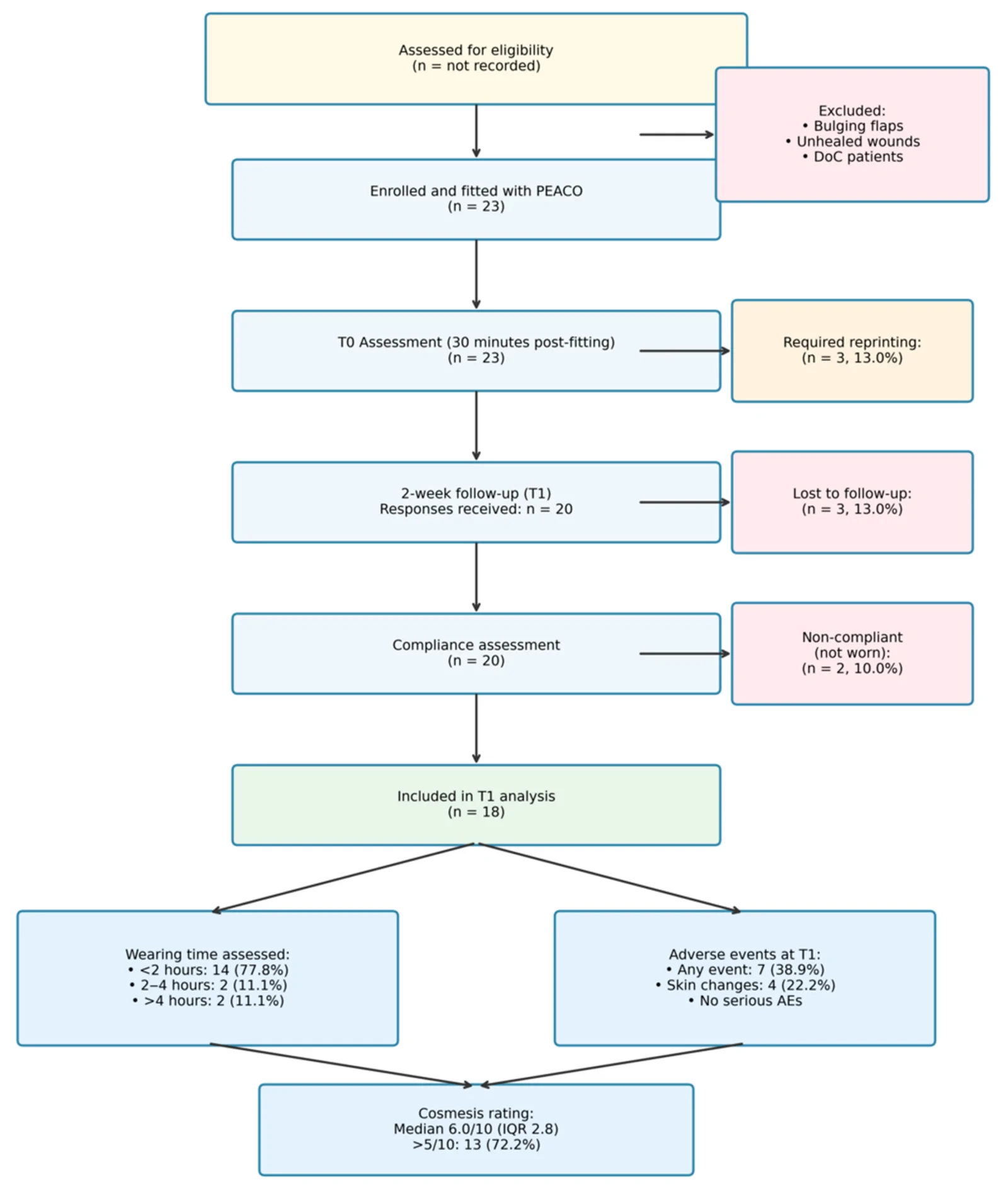
Patient flow diagram (*n* = 23).

The recruitment flow diagram is shown in Figure 3.

During the study period, all patients admitted post-decompressive craniectomy to the rehabilitation unit were consecutively screened for eligibility. Patients were excluded if they had bulging cranial flaps, unhealed surgical wounds, or disorders of consciousness. Of those screened, 23 consecutive eligible patients were fitted with PEACO and included in the analysis.

Altogether, 23 patients were fitted with current PEACO, including 14 males (60.9%), median (IQR) age 56.0 years (24.0), at median 67 (35.5) days post-DC; 13/23 (56.5%) with TBI and 10/23 (43.5%) with stroke (Table 1). At T0, primary reprinting was needed in 3/23 (13.0%) patients for poor initial fit (2) and post-fitting design modifications (1), hence a total of 26 PEACO (current) was printed for 23 patients (File S5, Table S2). Therapist-applied padding was noted in 4/23 (17.4%). Individual data for experimental and current versions are available on request. At T0, 18/23 (78.3%) fitted patients were confirmed compliant, 18/20 (90%) were compliant with responder feedback; 2/20 (10.0%) were non-compliant, with three patients of unknown status. At T0, 9/23 (39.1%) had therapist-observed mild, transient adverse events, with no reported surgical wound dehiscence or skin breakdown. At T1, among 18 respondents: 16/18 (88.9%) wore PEACO ≤4 h/day, 7/18 (38.9%) reported self-limiting discomfort or transient skin changes at T1, median cosmesis rating was 6.0/10. At T1 assessment, no documented falls with head impact occurred. (Refer Figure 3). Further analysis of post-discharge data revealed that a median (IQR) of 234 (247) days elapsed from DC to elective cranioplasty, representing a prolonged period where the cranial flap was unprotected with risk of secondary brain injury.

### 3.2. Follow-Up Data (T1)

The review of electronic medical records confirmed no incident reports of falls with head impact for any of the 23 PEACO-fitted patients during their inpatient rehabilitation stay. It was noted that one patient’s current admission for TBI was related to an unprotected DC cranial flap injury which occurred 506 days prior.

At T0, relative to 23 fitted patients, the reported device use rate was 18/23 (78.3%) among all fitted patients; among the 20 respondents, 18/20 (90.0%) confirmed device use, and 3/23 participants had unknown compliance status. The compliance status of three patients were unknown, two of three non-responders had been discharged to nursing homes compared to 2/20 (10%) of responders, suggesting that non-response was likely related to systemic care factors (e.g., lack of adequate assistance to don/doff PEACO) and may not be due to random factors (Refer to File S5, Table S3).

A conservative sensitivity analysis treating all three non-responders as non-compliant yielded an overall compliance rate of 18/23 (78.3%) (Refer to File S5, Table S3). Based on 20/23 digital responses received (File S5, Table S4), 18/20 (90%) had complete responder data. Non-responders had similar baseline characteristics to responders including median age (52.0 vs. 56.5 years), (refer to File S5, Table S3). Of these 20 respondents, 2/20 (10%) were non-compliant with PEACO wear (i.e., PEACO was not worn during the monitoring period of 2 weeks), leaving 18/20 (90.0%) at T1 with responder data (see Table 2). Table 3 shows individual self-reported adverse events recorded at T1. All were mild, self-limiting, and resolved with skin rest from PEACO.

### 3.3. Comparison of Follow-Up Data Between Experimental and Current PEACO

**Figure 4:**
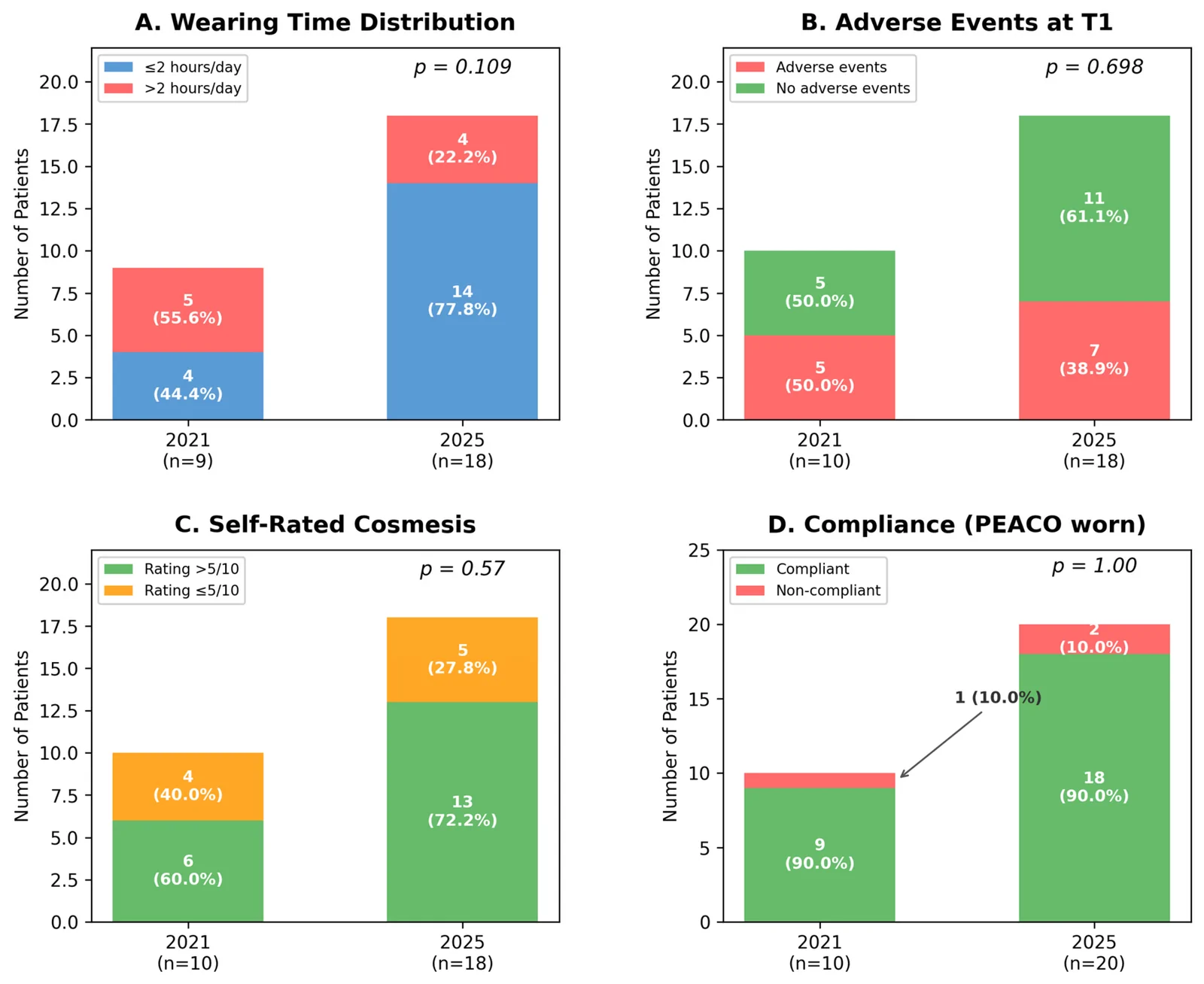
Four-panel comparison of experimental and current PEACO versions.

Comparing mild adverse event incidence of 5/10 (experimental) versus 7/18 (current), this corresponded to an absolute risk difference of −11.1 percentage points (95% CI =−43.3 to 23.5; *p* = 0.698, Fisher’s exact test), whilst self-rated cosmesis scores were >5/10 in most respondents in both cohorts. Non-compliance rates were similar between both cohorts (1/10, 10.0% [experimental] vs. 2/20, 10.0% [current], *p* = 1.00, Fisher’s exact test). Wearing time at up to 2 h/day was similar between groups (4/9, 44.4% [2021] vs. 14/18, 77.8% [2025], *p* = 0.109, Fisher’s exact test). Overall duration from decompressive craniectomy to cap fabrication was shorter in the current cohort (87.5 days [experimental] vs. 67 days [current], *p* = 0.189, Mann–Whitney U test). Adverse events (pressure, pruritus, and pain) were lower for current version (5/10, 50% [experimental] vs. 7/18, 38.9% [current]), and acceptability, based on an exploratory post hoc threshold selected to distinguish moderate-to-high from low acceptability, was higher (6/10, 60% [experimental] vs. 13/18, 72.2% [current], *p* = 0.57, Fisher’s exact test) (Refer to File S5, Table S1). These comparisons are summarised in Figure 4.

None of the observed differences reached statistical significance. However, the absence of statistical significance in these small samples should not be interpreted as evidence of no meaningful difference between versions; the study was not powered for such comparisons and the findings remain exploratory. Given the small sample sizes, methodological differences requiring variable harmonisation, and absence of statistically significant differences, these exploratory comparisons are presented descriptively and should not be interpreted as evidence of superiority of either version.

## 4. Discussion

### 4.1. General Findings

In this report, we describe the initial prescriptive clinical protocols and therapist-observed and PCRO of an inpatient clinical service for PEACO employing a 2-week minimally monitored protocol by rehabilitation therapists, embedded in the inpatient rehabilitation programme, as additional staff were not employed to run the PEACO clinical service. Data were retrospectively collected by a research assistant to assess adverse events and PCRO. During inpatient rehabilitation, the median admission FIM (IQR) was 29 (20) (Table 1), indicative of very severe disability (FIM < 40), while the median within-participant paired FIM change (IQR) (*n* = 21 with paired admission and discharge measurements) was 26, attesting to the benefit of inpatient rehabilitation in these 23 post-DC patients, wherein their FIM gains exceeded minimal clinically important differences (MCID) interchangeable thresholds for stroke (*n* = 10) and TBI (*n* = 13) at ∆22 (total FIM) [20,21].

### 4.2. Comparison of Compliance and PCRO for 2 Versions of PEACO

The simple descriptive comparisons between current PEACO with historical experimental version should be regarded as purely exploratory and speculative, given the small sample sizes in both cohorts, methodological differences in the two versions of the devices, outcomes needing harmonisation of outcome definitions, and non-significant results. Nevertheless, some similarities were noted in terms of compliance (>75%) and absence of serious adverse events defined as death, disability, wound infections, or skin dehiscence related to cap wearing for both versions over monitoring periods of 2 to 24 weeks.

The short follow-up period of 2 weeks arbitrarily, was intentional to balance staff productivity, recall ability of patients and carers and prior safety of historical cohort [13]. We acknowledge this duration precludes assessment of long-term durability and sustained compliance over the median 234-day interval to cranioplasty. Future studies should incorporate serial assessments at up to 6–12 months to characterise device performance, compliance, and symptoms throughout the pre-cranioplasty period.

The absence of documented falls with head impact or secondary TBI during the inpatient phase was likely due to vigilant fall precaution measures within neurorehabilitation wards. That said, the long-term protective capabilities attributable to PEACO or comparisons with other designs cannot be assumed with this report. Future studies should prospectively document fall events and unprotected cranial flap impact incidents throughout the entire period of PEACO use until cranioplasty. It was observed that one TBI from cranial flap trauma from a previous DC with unprotected cranial flap was prescribed PEACO. Despite the rarity of such reports, prevention remains key to reducing further morbidity in DC survivors. Apart from these reasons, Gustavsson et al. offered a fresh perspective on DC-related reduction in QOL and alteration in routines suffered by more independent patients without cranial bone protection prior to cranioplasty, thus PEACO could potentially enhance independence and activity levels for this category of patients in the months awaiting cranioplasty [22].

### 4.3. Comparison of Adverse Events and PCROs Between 2 Versions of PEACO

Noting differences in device materials and improved design for stability, independent donning/doffing and mass, any comparisons are purely exploratory, descriptive and speculative. Reprinting rate for poor fit was observed to be relatively low at 13% and we posit this disparity possibly to a higher complexity of design elements in the current PEACO; and a 3-week shorter time to fit post DC (current) vs. median 87.5 days [experimental] after DC. Compliance (10% for both versions), wearing times and cosmesis were largely similar without significant differences.

Future design iterations could explore colour customisation, reduction in visible strapping or mass reduction and integration with patient-selected headwear such as wigs or caps. These findings concur with previous reports which reported high levels of tolerability, acceptability, and compliance [12], underpinning the future design iterations could explore colour customisation status of 3D printing, which has rapidly transformed orthotics devices through precise customization based on patient anatomy, rapid and streamlined fabrication [1,23,24].

### 4.4. Comparison with Current Literature

Several studies have reported on their clinical utility, short-term tolerability, and patient–carer-reported outcomes (PCRO), albeit using closely monitored protocols [13,25]. A wide variety of materials, interfaces for attachments, follow-up durations and outcome measures for external protective cranial devices have been reported. Fernandez et al. [12] published a scoping review of innovative devices, where seven of the nine devices used 3D printing technology mirroring the contralateral intact skull, providing the fundamental evidence for multidisciplinary teams designing such devices to reduce risks associated with post-DC complications. Currently, 3D-printed external cranial caps are rapidly gaining recognition as a feasible temporary cranial protective device following DC, with emerging evidence on short-term tolerability

In terms of external interfaces with the cranium, various customised features and materials were employed to ensure interface and conformability to the external cranium, in the form of elastic band strapping, buckles, foam pads, non-adhesive materials, bio-adhesives, vacuum attachment devices, and valves, in association with allowances for cultural adaptations (e.g., worn under headwear). Regarding follow-up periods, the six case reports described ranged from 8 days to 4 months, while the cohort study by Chua et al. followed up 10 patients over 8 weeks [13]. Outcomes were highly variable ranging from global outcomes by Glasgow Outcome Scale (GOS) to mobility outcomes [12]. Most reports alluded to the high acceptability and lack of serious adverse events, while two case reports of plaster and vacuum devices indicated partial reversal of symptoms of Syndrome of Trephined Brain [12,25].

While our cohort achieved clinically meaningful FIM gains despite severe impairment expected of DC survivors, these functional improvements occurred during comprehensive inpatient rehabilitation and could not be attributed to PEACO wear. In contrast, a retrospective study comparing controls with external cranial orthoses with 8 h/day of wear, fitted early at <14 days post-DC, found a significant reduction in subdural effusion without skin issues although global outcomes between both groups were similar [25]. Ma et al. reported a retrospective series of 25 patients who received wig-based prosthetics compared to controls, with significantly increased self-assessed aesthetics ratings, quality of life ratings and increasing wearability from 2 to 5 h daily over a 12-week closely monitored protocol [25]. The weights of their devices were also comparable to the current 2025 PEACO (132 g Ma vs. 108 g PEACO), as well as approximately 40% who had at least one complaint of skin irritation, in concurrence with our findings of 39% with skin adverse events. However, unlike such closely monitored research protocols, the present case series reports a preliminary clinical pathway, pragmatic, efficient integration within routine rehabilitation practice of complex workflows through close collaborations across various disciplines, minimising needs for additional resources.

### 4.5. Other Considerations

The exclusion of patients with disorders of consciousness (DoC) merits discussion. While these individuals may benefit most from cranial protection due to the prolonged trajectory for functional and locomotor recovery, cranioplasty may be delayed due to perceived higher surgical and anaesthetic risk and relative lack of clinical guidelines for such populations. They also face the greatest challenges to PEACO self-monitoring, necessitating increased observation time from nurses or caregivers. Controversies also exist as to the higher institutionalisation rates for DoC patients, where increased recumbency and reduced staff monitoring for PEACO complications or compliance in nursing homes may alter the risk-benefit ratio for external cranial protection. Further studies are warranted for this subpopulation.

### 4.6. Global Health Perspectives

From a global health perspective, the burden of DC is substantial and growing. TBI affects an estimated 27 to 69 million individuals annually worldwide, with an estimated lifetime cost of USD 600,000 to 1.8 million per case [26]. The disease burden is disproportionately borne by low- and middle-income countries (LMICs), which experience approximately three times more TBI cases than high-income countries and 3–4-fold higher TBI-related mortalities [26,27,28,29]. The injury-related disease burden in LMICs substantially exceeds global averages when measured by disability-adjusted life years [30].

The following observations are presented as context for future research and do not constitute findings from this study. In our cohort, the median time from DC to definitive cranioplasty was 234 days (~8 months), representing a prolonged window of risk for various secondary post-DC complications. Given the substantial lifetime costs associated with TBI-related disability, estimated to be USD 76.5 billion for population benchmarks of direct and indirect economic costs and per-patient lifetime costs of severe TBI in the range of USD 600,000 to USD 1–2 million with inclusion of indirect costs [31,32], and mean total direct intramural costs for severe TBI of EUR 60,400 (IQR EUR 24,400–112,700) in European settings [33] DC and cranioplasty warrant future investigation [34,35]. The economic implications of secondary TBI during the vulnerable period between future health-economic evaluations are warranted to determine whether external cranial orthoses may reduce the burden of secondary TBI-related costs during the pre-cranioplasty period.

For context, generic sports helmets cost SGD 150–400 but are rarely routinely prescribed due to poor fit, mass and poor cosmesis, lacking anatomical customisation, while commercial medical-grade protective headgear costs SGD 160–350. PEACO thus represents a higher-priced option offering manufacturer-reported material properties, precise personalisation through CT-derived anatomical customization, improved comfort through low mass, ventilated materials in view of local humidity, and accommodation of individual scalp features unavailable in commercial alternatives. Comprehensive cost-effectiveness analyses of external cranial protection devices remain limited and were not performed in this study. The above cost figures and statements are presented solely as context for future economic evaluation, not as evidence of cost-effectiveness of PEACO.

### 4.7. Implementation Science Perspectives

The generalisability of PEACO programmes to diverse healthcare contexts warrants careful consideration. While our study was conducted in a well-resourced tertiary rehabilitation centre with on-site 3D printing capabilities, alternative implementation models may be necessary for settings with limited resources. Recent evidence suggests that affordable, patient-specific cranial devices can be produced using low-cost materials and open-source software, potentially enabling PEACO implementation in LMICs [36,37]. Collaborative networks between high-income and low-resource settings, following models such as the NIHR Global Health Research Group on Neurotrauma, could facilitate technology transfer and capacity building [12]. Furthermore, integration of cranial protection protocols into existing DC management guidelines, as advocated by the International Consensus Meeting on Decompressive Craniectomy [38], provides a rationale for future multicentre implementation studies.

Second, while the full PEACO pathway requires substantial infrastructure that may limit applicability in resource-constrained settings, the integration of clinical screening and monitoring within existing rehabilitation workflows, as demonstrated by our minimally monitored protocol, reduced the need for additional staffing within established tertiary centres. Third, the confirmed compliance rate of 78.3% (18/23) in our study, comparable to rates reported in other series [25]. From an implementation science perspective, several protocol factors merit consideration for broader adoption of the PEACO model described in this report. First, the minimally monitored model leveraging on existing staff demonstrates feasibility without additional personnel; while polymer resin materials and CT mirroring techniques are already employed for low-cost cranioplasty in LMICs [36,37], the full PEACO pathway requires CT imaging, specialised 3D printing infrastructure, technical post-processing, and therapist fitting, representing substantial resource requirements distinct from the low additional monitoring burden demonstrated in this study, suggesting acceptable initial adherence, though scalability will require larger multi-centre trials.

## 5. Limitations and Conclusions

### 5.1. Study Limitations

The following study limitations are highlighted: retrospective design, small single-centre sample, absence of a concurrent control group, variable >2 week follow-up period represented a fraction of time in contrast to the 234 days of needed use till cranioplasty, 13.0% (3/23) nonresponse rate (with unknown compliance status), caregiver-related selection bias, use of non-validated questionnaires, lack of objective wear monitoring, possible incomplete adverse-event detection and methodological differences from the historical cohort, lack of long-term follow-up data with regard to compliance, material degradation, delayed complications, secondary TBI and falls during the post-discharge phase; limited health-related quality of life and qualitative outcomes. To compare effects of two iterative research and service versions of PEACO, the use of harmonised disparate adverse events between two historical experimental and current versions over 4 years introduced technical and methodological differences and weakness; and was purely descriptive and exploratory. The absence of falls during the short follow-up period could not prove protection efficacy. We acknowledge the limitation of protective performance with isolated material impact resistance values. Longer follow-up periods with longer PEACO exposure will allow richer qualitative feedback as patients regain their societal roles. The categorical classification of wearing duration and binary definition of non-compliance represented coarse measures for this exploratory report. An essential safety requirement for an available caregiver and the exclusion of patients with disorders of consciousness could have introduced important selection bias; however, these criteria were mandatory to ensure adequate safety and outcome reporting.

Wearable sensors embedded in PEACOs could provide more nuanced adherence data and fall detection [12]; however, such additions would increase device cost and complexity, which may not be justified for a temporary cranial protective device intended for disposal after cranioplasty.

### 5.2. Conclusions

Findings from our study provide a detailed preliminary clinical implementation pathway conducted during inpatient brain injury rehabilitation. A minimally monitored inpatient PEACO clinical protocol achieved 18/23 (78.3%) compliance without serious adverse events (e.g., wound dehiscence/infection) across 23 decompressive craniectomy patients. Manageable minor adverse events in ~38% and short-term wearability were demonstrated. Successful integration within established tertiary centre workflows without additional personnel for monitoring, provides the initial framework for future studies on broader scalability, implementation and future evaluation of economic impact or cost-effectiveness. The pre-requirement of caregivers underscores the need for consistent home-based monitoring post-discharge due to participant vulnerability. The short follow-up period and absence of cranial trauma during the 2-week period cannot establish cranial protective efficacy, long-term safety, or superiority of either design, cost effectiveness or general scalability.

Close multidisciplinary collaboration between rehabilitation clinicians and 3D printing technicians, are critical factors for implementation success. With the advancement of technological development and publications related PEACO versions, larger, robust multicentre trials are clearly needed to demonstrate reproducibility of such preliminary outcomes. Further studies should report standardised patient qualitative outcomes and develop future standards for optimum materials and fabrication, in terms of strength, durability and mass, design interface, device regulation. Further research directions could include standardisation of materials, design interfaces, monitoring of adherence and outcome measures till cranioplasty to establish cranial protective capabilities over a long-term period and develop clinical practice guidelines for post-DC cranial protection devices.

## Figures and Tables

**Table 1:** Summary baseline demographic, clinical and PEACO characteristics at T0 (*n* = 23).

Variable	Median (IQR)	Frequency (*n*, %)
Age (years)	56.0 (24.0)	
Gender		
Male		14 (60.9)
Female		9 (39.1)
Race		
Chinese		18 (78.3)
Non-Chinese		5 (21.7)
Aetiology		
Traumatic Brain Injury		13 (56.5)
Stroke		10 (43.5)
Decompressive Craniectomy Side		
Left		12 (52.2)
Right		9 (39.1)
Bilateral frontal		2 (8.7)
Length of Stay, days (*n* = 22)		
ALOS	24.5 (13.2)	
RLOS	52.0 (23.0)	
FIM Admission (*n* = 22)		
Total FIM	28.5 (20.0)	
Motor FIM	18.0 (15.0)	
Cognitive FIM	8.5 (11.2)	
FIM Discharge (*n* = 21)		
Total FIM	68.0 (24.0)	
Motor FIM	48.0 (19.0)	
Cognitive FIM	16.0 (9.0)	
Discharge Destination		
Home		19 (82.6)
Nursing Home		4 (17.4)
Duration (Days)		
Craniectomy to PEACO Fit	67.0 (35.5)	
Admission to PEACO Fit	70.0 (35.5)	
Craniectomy to Cranioplasty (*n* = 15)	234 (247.0)	
Weight of PEACO (grams)	108 (27.5)	
Adverse events at T0		
Yes		9 (39.1)
No		14 (60.9)
Observed adverse events		
Skin Changes		5 (21.7)
PEACO Modifications		2 (8.7)
Pressure		2 (8.7)
Pain		1 (4.4)
Itch		0 (0.0)

PEACO Modifications (reheating and reprinting) are reported separately from clinical adverse events (skin changes, pressure, pain, itch). Legend: Incomplete records for Length of Stay (*n* = 22), FIM Admission (*n* = 22), FIM Discharge (*n* = 21). Missing cranioplasty data (*n* = 8): four declined, two lost to follow-up, one deceased, one pending post-discharge follow-up. Nine patients reported adverse events at T0; the sum of individual clinical adverse event categories (skin changes five, pressure two, pain one) totals eight symptom records because one patient reported both a clinical symptom and a technical modification event. PEACO Modifications (reheating two, reprinting three) are reported separately from clinical adverse events. Adverse event categories harmonised between cohorts as described in Methods. Abbreviations: ALOS, Acute Length of Stay; RLOS, Rehabilitation Length of Stay; FIM, Functional Independence Measure; PEACO, 3D-Printed Externally Applied Cranial Orthosis.

**Table 2:** Summary of Patient–Carer-Reported Outcome (PCRO) for subjective self-rated feedback at T1 for current PEACO (*n* = 18).

Variable	Median (IQR)	Frequency (*n*, %)
Wearing Duration (*n* = 18)		
0–1 h		14 (77.8)
2–4 h		2 (11.1)
>4 h		2 (11.1)
Comfort Scale (*n* = 18)		
Comfort (score/10)	8.0 (3.0)	
Appearance Scale (*n* = 18)		
Cosmesis (score/10)	6.0 (2.8)	
Reported Adverse events at 2 Weeks		
Yes		7 (38.9)
No		11 (61.1)
Reported Adverse events (*n* = 18)		
Skin Changes		4 (22.2)
Pressure		4 (22.2)
Itch		3 (16.7)
Pain		0 (0.0)

Legend: 18/20 respondents had worn the cap for 2 weeks. Adverse event categories harmonised between experimental and current PEACO versions as described in Methods.

**Table 3:** Individual self-reported Patient–Carer-Reported Outcome (PCRO) for adverse events (PEACO current) at T1 (*n* = 18).

Participant No.	Pressure	Skin Changes	Itch	Pain
C01	No	No	No	No
C02	No	No	No	No
C06	Yes	Yes	No	No
C07	No	No	No	No
C08	Yes	Yes	Yes	No
C09	Yes	No	No	No
C10	No	No	No	No
C11	No	No	Yes	No
C13	No	No	No	No
C14	No	Yes	No	No
C15	No	No	No	No
C17	Yes	No	Yes	No
C18	No	No	No	No
C19	No	No	No	No
C20	No	No	No	No
C21	No	No	No	No
C22	No	Yes	No	No
C23	No	No	No	No

Legend: Adverse event categories harmonised between cohorts as described in Methods. Missing participants C05 and C12 did not wear their caps, and C03, C04, and C16 were non-responders.

## Data Availability

Individual patient data are available on request from the corresponding author. The data are not publicly available due to privacy restrictions.

## Supplementary Materials

The following supporting information can be downloaded at: https://www.mdpi.com/article/10.3390/healthcare14203361/s1, File S1: Ethics approval and study chronology (Figure S1: Study chronology); File S2: CT acquisition parameters; File S3: Patient counselling documents (Figure S2: Patient information leaflet; Figure S3: Patient care instructions leaflet); File S4: Data collection form (Figure S4: FormSG post-evaluation form); File S5: Non-responder profile, technical modification and confidence interval analysis (Table S1: 95% confidence intervals for the main proportions and effect estimates for the comparison between the experimental (2021) and current (2022) cohorts; Table S2: Individual technical modifications made to PEACO (*n* = 23); Table S3: Baseline characteristics of responders and non-responders; Table S4: Individual dates of FormSG request and of T0 and T1 assessments (*n* = 23)).

## Abbreviations

The following abbreviations are used in this manuscript:

DC	Decompressive Craniectomy
DoC	Disorders of Consciousness
FIM	Functional Independence Measure
IQR	Interquartile Range
LMIC	Low- and Middle-Income Countries
MCID	Minimal Clinically Important Difference
PEACO	3D-Printed Externally Applied Cranial Orthosis
PCRO	Patient–Carer-Reported Outcome
QOL	Quality of Life
TBI	Traumatic Brain Injury
TTSH	Tan Tock Seng Hospital
