# Supplementary material for: Preliminary Feasibility Implementation of Post-Decompressive Craniectomy 3D-Printed Externally Applied Cranial Orthosis (PEACO) During Inpatient Rehabilitation: A Retrospective Cohort Study Reporting Short-Term Tolerability and Patient/Caregiver-Reported Feedback
Source: Healthcare (Basel). 2026 Oct 9;14(20):3361. doi: 10.3390/healthcare14203361 (PMC13703988; doi:10.3390/healthcare14203361)
Supplement: Supplementary file 1 [file healthcare-14-03361-s001.zip › healthcare-4509210-supplementary-File_S2.pdf]

CT Acquisition Parameters

Patient Positioning

Parameter	Setting
Patient Position	Head First Supine (HFS)
Head Alignment	Frankfurt Plane parallel to gantry
Immobilization	Head holder with foam support
Scan Coverage	Vertex to below skull base (foramen magnum)

Acquisition Parameters

Parameter	Proposed Protocol
Scan Mode	Helical (Spiral) CT
Tube Voltage (kVp)	120 kVp
Tube Current (mAs)	Automatic Exposure Control or 200-300 mAs equivalent
Rotation Time	1.0-1.2 s
Pitch	0.55-1.0
Detector Collimation	0.6 mm
Total Collimation Width	38.4 mm (scanner dependent)
FOV	220-250 mm (head only)
Matrix Size	512 × 512
Gantry Tilt	0°
Reconstruction Diameter	220-250 mm

Reconstruction Parameters

Bone Algorithm (For 3D Printing)

Parameter	Setting
Slice Thickness	0.5-1.5 mm

Slice Interval	0.5-1.5 mm
Reconstruction Kernel	Sharp Bone Kernel (e.g. H70 / Br64 / B70 equivalent)
Matrix	512 × 512
Pixel Size	≤ 0.5 mm

Image Export Requirements

Parameter	Setting
Format	DICOM
Compression	None
Reconstruction Planes	Axial
Additional Reconstructions	Coronal, Sagittal
Anonymization	Required for research datasets

CT Acquisition Protocol: Non-contrast cranial CT examinations will be acquired using a multidetector CT scanner with isotropic high-resolution imaging parameters optimized for three-dimensional reconstruction. Scans will be obtained at 120 kVp with submillimeter detector collimation (≤0.6 mm) and reconstructed using a dedicated bone algorithm with slice thickness ≤0.6 mm. DICOM datasets will be exported for image segmentation, computer-aided design, and 3D printing workflow development. The protocol is designed to achieve high anatomical accuracy while maintaining compliance with routine clinical imaging standards.
