# Supplementary material for: Preliminary Feasibility Implementation of Post-Decompressive Craniectomy 3D-Printed Externally Applied Cranial Orthosis (PEACO) During Inpatient Rehabilitation: A Retrospective Cohort Study Reporting Short-Term Tolerability and Patient/Caregiver-Reported Feedback
Source: Healthcare (Basel). 2026 Oct 9;14(20):3361. doi: 10.3390/healthcare14203361 (PMC13703988; doi:10.3390/healthcare14203361)
Supplement: Supplementary file 1 [file healthcare-14-03361-s001.zip › healthcare-4509210-supplementary-File_S3.pdf]

Completely Customised Cranial Cap (C4)

Overview

Patients who have severe stroke or a traumatic brain injury may undergo a life-saving operation, called "*decompression craniectomy*". In this surgery, a portion of the patient's skull bone has been removed, leaving a bone defect in the skull which offers little protection for the underlying brain.

The medical team will replace the missing skull bone several weeks or months later. During this period, the patient will potentially be at risk of secondary brain injury in case of a fall. As such, patients are usually recommended to wear rigid sports helmets as standard temporary protection. However, both types of helmets are bulky and uncomfortable to wear for long durations due to Singapore's tropical heat and humidity.

TTSH's Solution – Completely Customised Cranial Cap (C4)

The completely customised cranial cap (C4) was developed in 2019 to 2020 from a joint collaboration between TTSH Rehabilitation Centre and National Neuroscience Institute's (NNI) Neurosurgery Department. It is individually created using 3D printing design technology and is proven to be a safe and acceptable solution.

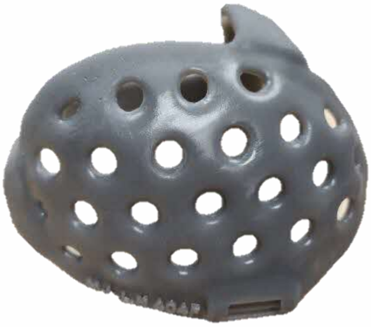

A 3D-printed Completely Customised Cranial Cap

Benefits of Using C4

As compared to standard helmets, there are several benefits of using a completely customised cranial cap.

- Custom fitted to the wearer as it is modelled precisely according to their intact skull
- It is light weight (200-250 grams)
- Ventilated material is used over the cap
- Improved comfort

How Is C4 Prescribed

- Before you are prescribed a C4, you will go through strict screening and clearance by your medical team. Including an assessment of your surgical wound healing, CT scans of the brain, and your verbal consent.
- Each C4 takes one to two weeks to print and customise. It will also require fitting and strapping onto your scalp by experienced therapists, followed by a 30-minute observation period.
- You may wear the C4 for as long as you have a skull defect.

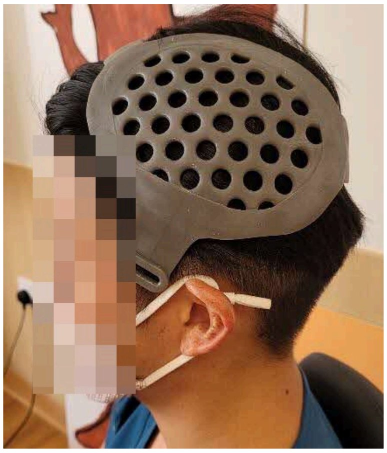

A patient wearing a Cranial Cap

Caring for Your C4

Your medical team will provide detailed instructions on the use and care for your C4. Please follow all instructions.

If you have any enquiries, please contact the following hotlines:
6889 4580 (TTSH CART), 6843 5309 (TTSH ICH Ward 9G), 6357 7095 (NNI Clinic)

Caring for your Completely Customised Cranial Cap (C4)

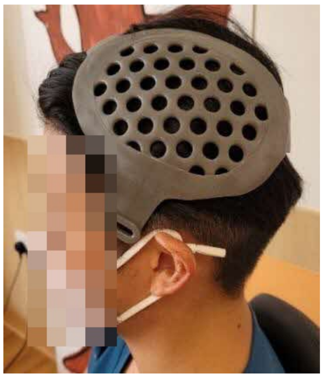

Above: A patient wearing a Cranial Cap

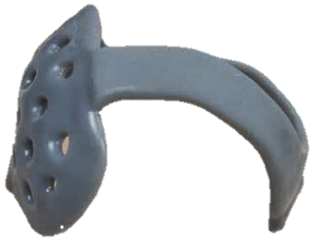

The C4 is a medical Head Protection Device that is 3D-printed according the unique CT image of your skull and fitted with customised rigid and soft straps.

Please refer to the following instructions for information on use and of your C4.

1. Cleaning Instructions

A. Cleaning the strap
- Wipe daily with damp cloth or wet-wipes
- Use mild soap to wash the strap only if it is badly soiled
- Ensure strap is completely dry before use

B. Cleaning the head protection device
- Wipe daily with alcohol or wet-wipes to keep it clean
- Use mild soap to wash the head protection device regularly
- Wipe dry with a soft cloth
- Avoid placing in extremely hot or cold water

2. Recommended Activities for Wearing of the Head Protection Device:
- Walking outdoors
- Walking indoors on level ground and climbing stairs
- During home exercises such as balance training
- During rehabilitation therapies (e.g. physiotherapy/ occupational therapy) in clinic or at Day Rehab Centres
- While travelling by car or public transport

During this period, you should AVOID driving or doing any form of contact sports or high impact activities (e.g. football, rugby, jumping from height, cycling) until your treatment team gives you the clearance to do so.

3. Suggested C4 Wearing Schedule for First-time Users:
- Day 1: wear for 30 minutes
- Day 2: wear for 1 hour
- Day 3: wear for 2 hours
- Do wear your head protection device as above until you undergo cranioplasty, which is a surgical replacement of a skull defect.

4. STOP and REMOVE the C4 immediately if You Notice the Following Signs
- Skin break down, redness, bleeding, discharge, pus from your surgical scar. Please contact your doctor immediately.
- Skin imprints around C4 site
- Severe discomfort or pain at surgical scar/ C4 contact
- Allergic reaction to the C4, i.e. severe itch/ rashes/ swelling/ redness

DEPARTMENT OF REHABILITATION MEDICINE
CART, Annex 2 Building, Level 1 | Tel: 6889 4580

NATIONAL NEUROSCIENCE INSTITUTE
NNI Block, Level 1 | Tel: 6357 7095

Tan Tock Seng HOSPITAL
National Healthcare Group

PECC-REH-ED-2023-1592-v1
