# Supplementary material for: Preliminary Feasibility Implementation of Post-Decompressive Craniectomy 3D-Printed Externally Applied Cranial Orthosis (PEACO) During Inpatient Rehabilitation: A Retrospective Cohort Study Reporting Short-Term Tolerability and Patient/Caregiver-Reported Feedback
Source: Healthcare (Basel). 2026 Oct 9;14(20):3361. doi: 10.3390/healthcare14203361 (PMC13703988; doi:10.3390/healthcare14203361)
Supplement: Supplementary file 1 [file healthcare-14-03361-s001.zip › healthcare-4509210-supplementary-File_S4.pdf]

Feasibility, safety and patient reported outcomes of 3D-Printed Externally-applied Cranial Orthosis (PEACO) after decompression craniectomy in clinical settings: A retrospective comparison study of monitored versus unmonitored protocols

DSRB ref: 2025/1218

Data Collection Form

List of variables:

1. Participant ID
2. Gender
3. Age
4. NRIC (last 3 digit)
5. Race
6. "Diagnosis
7. (TBI, Haemorrhage, Infarct, SAH)"
8. Detailed Diagnosis
9. Unilateral lesion (Y/N)
10. Bilateral lesion (Y/N)
11. Site of lesion (R/L)
12. Date of admission
13. Date of craniectomy
14. Duration (craniectomy - admission)
15. Cranioplasty (Y/N)
16. Date of cranioplasty
17. Duration (Cranioplasty-Craniectomy)
18. Date of rehab admission
19. Date of rehab discharge
20. Acute LOS
21. Rehab LOS
22. Date of CT stereotaxis
23. Glasses (Y/N)
24. Date of order (FormSG)
25. CD available date:
26. CD pickup date:
27. Cranial Cap (CC) available date:
28. Processing time (CC ready date - CD pickup)
29. CC weight
30. Strapping date:

31. Any side effects? (Y/N)
    a. If yes, specify - pain, redness, blister, imprint etc
32. Reheat (Y/N)
33. Reprint (Y/N)
34. 2-weeks follow-up date:
35. Follow-up FormSG date

FormSG post evaluation form

https://form.gov.sg/645d1265337d540012556ce1

1) Date of response:
2) Initials & last 3 digit of NRIC
3) Have you worn your Cranial Cap in the past 2 weeks?
4) Approximately, how many hours of wear per day including rest breaks?
    a) 0-1H
    b) 2-4H
    c) > 4H
5) Did you experience any of the following after 2 hours of wear? (Indicate more than one)
    a) Pain
    b) Discomfort
    c) Wound discharge
    d) Redness
    e) Skin markings
    f) None experienced
6) Please rate the comfort of your cranial cap from:
    a) Very uncomfortable (0) to Very comfortable (10)
7) Please rate the appearance of your cranial cap
    a) Unacceptable (0) to Very Acceptable (10)
8) Please rate the overall service of the Cranial Cap staff who attended to you:
    a) Satisfactory
    b) Neutral
    c) Unsatisfactory
9) Would you recommend the CC to others with the same condition:
    a) Yes
    b) No
    c) Neutral

Tan Tock Seng HOSPITAL

Cranial Cap Post evaluation form

Please complete this form after 2 weeks of wearing your cranial cap.
This will take about 5 minutes to complete and can be done by the patient or next of kin.

Please answer all questions

1. Date

dd/mm/yyyy

2. Initials & last 3 digit of NRIC

3. Have you worn your Cranial Cap in the past 2 weeks?

✕ No | ✓ Yes

4. Approximately, how many hours of wear per day including rest breaks?

- ○ 0-1H
- ○ 1-2H
- ○ 2-4H
- ○ >4H

5. Did you experience any of the following after 2 hours of wear? (Indicate more than one)

- ☐ Pain
- ☐ Discomfort
- ☐ Wound discharge
- ☐ Redness
- ☐ Skin markings
- ☐ None experienced

6. Please rate the comfort of your cranial cap from:

Very uncomfortable (0) to Very comfortable (10)

☆ ☆ ☆ ☆ ☆ ☆ ☆ ☆ ☆ ☆

7. Please rate the appearance of your cranial cap

Unacceptable (0) to Very Acceptable (10)

☆ ☆ ☆ ☆ ☆ ☆ ☆ ☆ ☆ ☆

8. Please rate the overall service of the Cranial Cap staff who attended to you:

- ○ Satisfactory
- ○ Neutral
- ○ Unsatisfactory

9. Would you recommend the CC to others with the same condition:

- ○ Yes
- ○ No
- ○ Neutral
