# Supplementary material for: Preliminary Feasibility Implementation of Post-Decompressive Craniectomy 3D-Printed Externally Applied Cranial Orthosis (PEACO) During Inpatient Rehabilitation: A Retrospective Cohort Study Reporting Short-Term Tolerability and Patient/Caregiver-Reported Feedback
Source: Healthcare (Basel). 2026 Oct 9;14(20):3361. doi: 10.3390/healthcare14203361 (PMC13703988; doi:10.3390/healthcare14203361)
Supplement: Supplementary file 1 [file healthcare-14-03361-s001.zip › healthcare-4509210-supplementary-File_S5.pdf]

Table S1a. Confidence Intervals (CI) calculations and values (n=23)

Main Current Cohort Proportions

Outcome	Calculation	%	Wilson 95% CI
Confirmed compliance, full cohort	18/23	78.3%	58.1–90.3%
Compliance among respondents	18/20	90.0%	69.9–97.2%
Adverse events at T0	9/23	39.1%	22.2–59.2%
Adverse events at T1	7/18	38.9%	20.3–61.4%
Reprinting	3/23	13.0%	4.5–32.1%
Serious adverse event/wound dehiscence	0/23	0%	0–14.3%

Confirmed compliance was 18/23 (78.3%; 95% CI 58.1–90.3%).

Table S1b. Confidence Intervals (CI) calculations and values (n=23)

Between Cohort Comparisons

Outcome	Experimental	Current	Risk difference* (95% CI)
Non-compliance	1/10 (10.0%)	2/20 (10.0%)	0.0 pp (−31.3 to 21.7)
Wearing ≤2 h/day	4/9 (44.4%)	14/18 (77.8%)	+33.3 pp (−3.6 to 62.1)
Adverse events	**5/10 (50.0%)**	7/18 (38.9%)	−11.1 pp (−43.3 to 23.5)
Cosmesis >5/10	6/10 (60.0%)	13/18 (72.2%)	+12.2 pp (−20.5 to 44.8)

Table S2. Individual technical modifications made to PEACO (n=23)

Participant No.	Reheat	Reprint
**C01**	No	Yes
**C02**	No	No
**C03**	No	No
**C04**	No	No
**C05**	No	No
**C06**	Yes	No
**C07**	No	No
**C08**	No	No
**C09**	No	No
**C10**	No	Yes
**C11**	No	No
**C12**	No	Yes
**C13**	No	No
**C14**	No	No
**C15**	No	No
**C16**	No	No
**C17**	No	No
**C18**	Yes	No
**C19**	No	No
**C20**	No	No
**C21**	No	No
**C22**	No	No
**C23**	No	No

Table S3. Comparison of Baseline Characteristics Between Responders and Non-Responders at T1

**Variable**	**Responders (n=20)**	**Non-Responders (n=3)**
Age, median (IQR) years	56.5 (24.3)	52.0 (13.0)
Male, n (%)	13 (65.0)	1 (33.3)
Aetiology, n (%)		
- Traumatic Brain Injury	12 (60.0)	1 (33.3)
- Stroke	8 (40.0)	2 (66.7)
Discharge Destination, n (%)		
- Home	18 (90.0)	1 (33.3)
- Nursing Home	2 (10.0)	2 (66.7)

Legend: IQR, interquartile range. Non-responders were lost to follow-up; two of three had been discharged to nursing homes, suggesting non-response was related to logistical factors rather than systematic differences in PEACO experience.

Table S4. Individual data of Formsg request, clinical service implementation and assessment dates (n=23)

Participant No.	Date of Formsg Request	Date of T0 Assessment (Clinical service)	Date of T1 Assessment
**C01**	16-Nov-22	29-Nov-22	21-Dec-22
**C02**	23-Dec-22	06-Jan-23	12-Jan-23
**C03**	01-Feb-23	17-Mar-23	Lost to follow-up
**C04**	01-Feb-23	24-Feb-23	Lost to follow-up
**C05**	13-Apr-23	28-Apr-23	27-Jul-23
**C06**	03-May-23	22-May-23	26-Jul-23
**C07**	20-Jun-23	07-Jul-23	26-Jul-23
**C08**	20-Jun-23	07-Jul-23	08-Aug-23
**C09**	17-Jul-23	02-Aug-23	25-Aug-23
**C10**	29-Sep-23	16-Oct-23	19-Dec-23
**C11**	17-Oct-23	30-Oct-23	19-Dec-23
**C12**	13-Oct-23	04-Dec-23	29-Dec-23
**C13**	12-Jan-24	15-Jan-24	14-Feb-24
**C14**	12-Jan-24	26-Jan-24	10-May-24
**C15**	19-Jan-24	25-Jan-24	02-Apr-24
**C16**	30-Jan-24	20-Feb-24	Lost to follow-up
**C17**	04-Sep-24	18-Sep-24	03-Oct-24
**C18**	13-Nov-24	21-Nov-24	03-Jan-25
**C19**	17-Apr-25	25-Apr-25	09-Jun-25
**C20**	12-Jun-25	24-Jun-25	18-Jul-25
**C21**	15-Aug-25	27-Aug-25	09-Sep-25
**C22**	03-Oct-25	17-Oct-25	04-Nov-25
**C23**	28-Oct-25	07-Nov-25	20-Nov-25
